# Evaluation of the wing cell contour to distinguish between *Stomoxys bengalensis* and *Stomoxys sitiens* (Diptera: Muscidae) using outline-based morphometrics

**DOI:** 10.1016/j.crpvbd.2024.100204

**Published:** 2024-07-25

**Authors:** Thekhawet Weluwanarak, Tanawat Chaiphongpachara, Tanasak Changbunjong

**Affiliations:** a The Monitoring and Surveillance Center for Zoonotic Diseases in Wildlife and Exotic Animals (MoZWE), Faculty of Veterinary Science, Mahidol University, Nakhon Pathom, 73170, Thailand; b Department of Public Health and Health Promotion, College of Allied Health Sciences, Suan Sunandha Rajabhat University, Samut Songkhram, 75000, Thailand; c Department of Pre-Clinic and Applied Animal Science, Faculty of Veterinary Science, Mahidol University, Nakhon Pathom, 73170, Thailand

**Keywords:** Geometric morphometrics, Internal cell, Landmarks, Outlines, Stomoxyinae, Vector

## Abstract

The blood-sucking flies of the genus *Stomoxys* Geoffroy, 1762 (Diptera: Muscidae) are significant ectoparasites that can cause irritation and transmit pathogens to both animals and humans. Within the genus *Stomoxys*, two species, *Stomoxys bengalensis* and *Stomoxys sitiens*, have similar morphology and coexist in the same habitat. Accurate species identification of these flies is crucial for understanding disease vectors and implementing effective control measures. In this study, we assessed the effectiveness of outline-based geometric morphometrics (GM) by analyzing the wing cell contour of the first posterior cell (R_5_) to distinguish between species and sexes of *S. bengalensis* and *S. sitiens*. Our results demonstrate that the outline-based GM method is highly effective in distinguishing between species and sexes of these flies based on contour shape, with accuracy scores ranging from 90.0% to 97.5%. Therefore, outline-based GM emerges as a promising alternative to landmark-based GM or as a supplementary tool in conjunction with traditional morphology-based methods for species identification.

## Highlights

• *Stomoxys bengalensis* and *Stomoxys sitiens* were examined using outline-based geometric morphometrics.
• The contour of the first posterior wing cell (R_5_) effectively distinguishes between fly species and sexes based on its shape.
• Outline-based geometric morphometrics provides a promising alternative method for species identification.

## Graphical abstract

**Figure:**
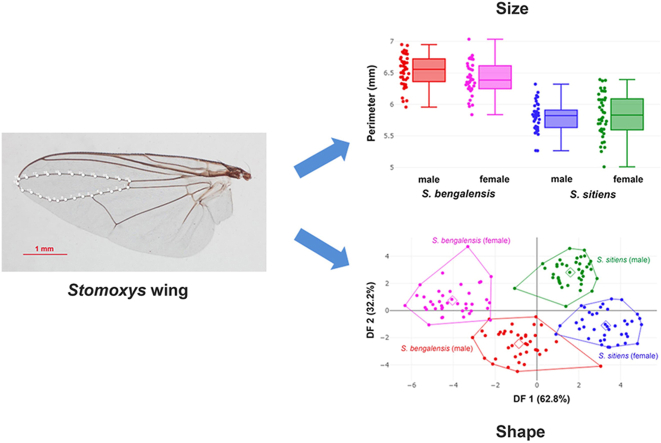

## 1 Introduction

Blood-sucking flies of the genus *Stomoxys* Geoffroy, 1762 (Muscidae: Stomoxyinae) are recognized as pests of veterinary and medical importance. They are not only a nuisance to humans and animals but also significantly impact animal health and productivity (Zumpt, 1973; Taylor et al., 2012). Currently, 18 species of the genus *Stomoxys* have been described, most of which are found in the Afrotropical realm. Additionally, some species are also found in the Oriental region and on Réunion Island (Duvallet and Hogsette, 2023). The cosmopolitan species *Stomoxys calcitrans*, also known as the stable fly, is well-known and has been reported as an important vector for various animal pathogens, including bacteria, helminths, protozoans, and viruses (Baldacchino et al., 2013). In Thailand, six species of *Stomoxys* have been identified, *S. bengalensis*, *S. calcitrans*, *S. indicus*, *S. pullus*, *S. sitiens*, and *S. uruma*, along with their respective distributions (Tumrasvin and Shinonaga, 1978; Muenworn et al., 2010; Changbunjong et al., 2012, 2023). Among these species, *S. calcitrans* has been documented as the most prevalent (Muenworn et al., 2010; Changbunjong et al., 2012), playing a significant role in disease transmission, particularly in the spread of trypanosomosis or surra in horses, cattle, and buffaloes (Desquesnes et al., 2013), anaplasmosis in cattle and buffaloes (Saetiew et al., 2018), and lumpy skin disease in cattle (Arjkumpa et al., 2022). Furthermore, Phetkarl et al. (2023) suggested that *S. sitiens* might be a potential vector for *Theileria* sp. in horses, as the parasite’s DNA was detected in the flies.

**Fig. 1:**
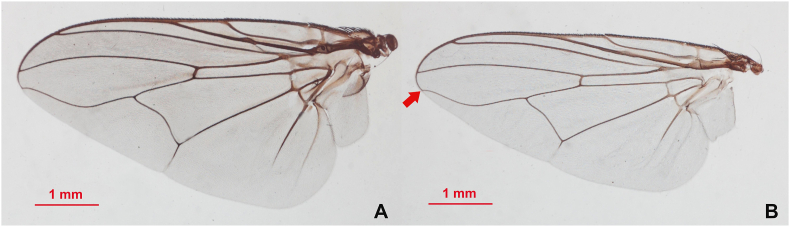
Wings of *Stomoxys bengalensis* (**A**) and *Stomoxys sitiens* (**B**). The pattern of the wing veins r_4+5_ (4th and 5th radial veins) (*arrow*) is used to distinguish between species.

Traditionally, distinguishing between species within the genus *Stomoxys* has been based on morphological traits such as body pattern and color, frons width, wing venation pattern, genital morphology, and leg hairs (Zumpt, 1973). These characteristics have helped identify and characterize the species, as well as differentiate between closely related ones. However, traditional morphological methods can be subjective and may not adequately distinguish between species with similar morphology, especially when specimens are unclear or lack all relevant morphological characters. Among the six *Stomoxys* species found in Thailand, *S. sitiens* and *S. bengalensis* have similar abdominal patterns (Zumpt, 1973). These species can be differentiated by their wing venation; in *S. sitiens*, the apex of the media is slightly proximal to the apex of the r_4+5_ (4th and 5th radial veins), while in *S. bengalensis*, it is positioned almost directly beneath the apex of r_4+5_ (Fig. 1). Moreover, male *S. bengalensis* have elongated ventral hairs on the hind femur, a characteristic absent in male *S. sitiens*. However, this key characteristic cannot be used to identify female flies. Therefore, if crucial morphological characteristics are damaged or lost, accurate identification could prove challenging.

Accurate species identification of *Stomoxys* flies is essential for identifying target vectors, which is crucial for developing effective vector control strategies. Therefore, molecular and geometric morphometric (GM) methods have emerged as valuable tools for precise species identification (Changbunjong et al., 2016a, 2016b, 2023). These techniques allow researchers to analyze genetic or phenotypic markers and then compare them with reference sequences or GM data, enabling precise species identification when morphological features are ambiguous.

Geometric morphometrics is a powerful tool for quantifying and analyzing size and shape variation among biological specimens. It involves capturing the geometry of biological forms using landmarks, outlines, or semi-landmarks, which are specific points identified on the structures of interest. The data are then analyzed using mathematical techniques such as Procrustes superimposition, principal components analysis, and discriminant analysis to visualize and interpret complex patterns of shape variation among specimens (Dujardin and Dujardin, 2019). This method offers simplicity, speed, and cost-effectiveness compared to molecular techniques, which may require expensive reagents and equipment, or morphological approaches that demand skilled personnel and substantial time investments (Dujardin, 2008; Changbunjong et al., 2023). The GM has emerged as a valuable tool for species differentiation among various insect groups, particularly significant vectors. This method has proven effective in distinguishing between species of mosquitoes (De Souza et al., 2020; Simões et al., 2020; Martinet et al., 2021; Chaiphongpachara et al., 2022a; Laojun et al., 2023), fleas (Chotelersak et al., 2024), blow flies (Sontigun et al., 2017), sand flies (Giordani et al., 2017), tsetse flies (Kaba et al., 2017), and horse flies (Changbunjong et al., 2021). For *Stomoxys* flies, GM has demonstrated the ability to distinguish among the three closely related species, *S. indicus*, *S. pullus*, and *S. uruma*, by analyzing landmarks and outlines of the external wing contour (Changbunjong et al., 2016a). Recently, Changbunjong et al. (2023) demonstrated the high effectiveness of GM in distinguishing among other *Stomoxys* species (*S. calcitrans*, *S. sitiens*, and *S. bengalensis*) using a landmark-based approach.

Recent reports suggest that wing cell contours are effective in identifying certain insects, such as disease-vector mosquitoes (Laojun et al., 2024). These contours, formed by a network of wing veins that create closed “cells” within the membrane, reflect the crucial role of flight in these insects (Parchem et al., 2007). An outline-based GM supports this analysis by examining differences in each contour using pseudo-landmarks (Dujardin et al., 2014). However, the effectiveness of using wing cell contours to distinguish among *Stomoxys* species remains in doubt, especially between *S. sitiens* and *S. bengalensis*, which have wing venation patterns long recognized as valuable taxonomic characters. The contour of the first posterior cell (R_5_) in the wings of *Stomoxys*, a large wing cell situated in the middle of the wing and connected to other significant cells, is particularly noteworthy. Given its significance, this cell could be crucial for distinguishing between morphologically similar and traditionally difficult-to-differentiate *Stomoxys* species, such as *S. sitiens* and *S. bengalensis*. Thus, studying this cell could improve our understanding of the morphological differences between *Stomoxys* species.

In this study, the contour of the wing cell, specifically the first posterior cell (R_5_), was used to assess its effectiveness in distinguishing between *S. bengalensis* and *S. sitiens* using outline-based GM. The results of this study provide an alternative method for distinguishing between these fly species.

## 2 Materials and methods

### 2.1 Fly collection and species identification

**Fig. 2:**
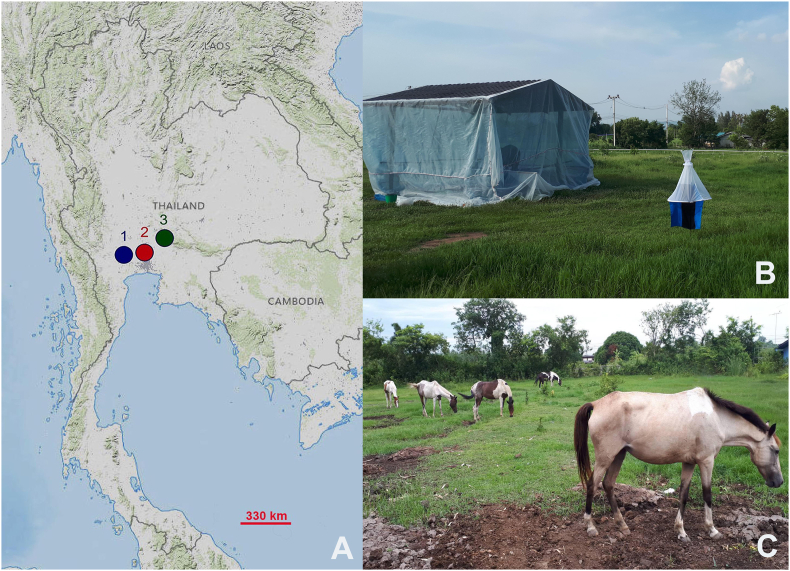
**A** Map of fly collection sites used in this study: Nakhon Pathom (1), Pathum Thani (2), and Saraburi (3). **B** Vavoua trap used for fly collection. **C** Animal hosts at the collection sites. The map was generated using the USGS National Map Viewer (https://www.usgs.gov/tools/national-map-viewer), accessed on May 10, 2024.

Specimens of both sexes of *S. bengalensis* and *S. sitiens* were collected from animal farms in three central Thai provinces, Nakhon Pathom, Pathum Thani, and Saraburi, using five Vavoua traps (Laveissiere and Grebaut, 1990) (Fig. 2, Table 1). All traps were randomly placed near animal hosts and enclosures and operated for four consecutive days during daylight hours (6:00–18:00 h). All specimens captured were euthanized by freezing at −10 °C, individually placed in 1.5 ml microcentrifuge tubes, and then transported to the Vector-Borne Diseases Research Unit at the Faculty of Veterinary Science, Mahidol University, Nakhon Pathom, Thailand.

**Table 1:** Collection sites and sample sizes (*n*) for *Stomoxys bengalensis* and *Stomoxys sitiens* used in the outline-based geometric morphometric analysis.

Species	Month/Year	Province (Coordinates)	*n*
*S. bengalensis*	May 2022	Nakhon Pathom (14°01′10″N, 99°57′37″E)	Male: 20; Female: 20
	July 2022	Pathum Thani (14°04′46″N, 100°31′51″E)	Male: 20; Female: 20
*S. sitiens*	May 2022	Nakhon Pathom (14°01′10″N, 99°57′37″E)	Male: 20; Female: 20
	November 2022	Saraburi (14°28′39″N, 101°05′33″E)	Male: 20; Female: 20
Total			160

Specimens with clear morphological characteristics were selected for species identification based on Zumpt’s descriptions and taxonomic keys (Zumpt, 1973), using a stereomicroscope (Nikon SMZ745; Nikon Corp., Tokyo, Japan). Zumpt (1973) provided separate keys for identifying male and female specimens of *Stomoxys*. Therefore, specimens designated for GM analysis were examined separately based on their sex and species identification (Changbunjong et al., 2023). Subsequently, these specimens were securely stored in a freezer at −20 °C pending further GM analysis.

### 2.2 Specimen preparation

The left wings of male and female *S. bengalensis* and *S. sitiens* were carefully dissected from the thorax using a sterile blade and mounted on microscope slides using Hoyer’s medium (Changbunjong et al., 2016a). Each wing slide was then photographed using a digital camera connected to a stereomicroscope (Nikon AZ 100; Nikon Corp., Tokyo, Japan), with a 1-mm scale-bar added to each image. A total of 160 wing images (Table 1) were captured, comprising 80 wings each of *S. bengalensis* and *S. sitiens*, which were then subjected to outline-based GM analysis.

### 2.3 Wing geometric morphometric analyses

#### 2.3.1 Outline digitization and digitization error

**Fig. 3:**
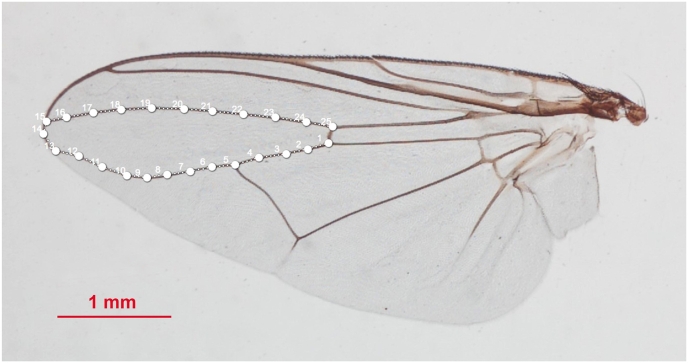
Digitized contour (white spots and numbers) of the first posterior cell (R_5_) used for outline-based geometric morphometric analysis.

The contour of the first posterior cell (R_5_) of the wings was digitized by manually outlining it, without requiring an equal number of points (also called pseudo-landmarks) for each individual. The contour was digitized consistently using the same starting and ending points (Fig. 3, Supplementary Table S1). This specific wing cell was selected because it has taxonomic features that aid in distinguishing between *S. bengalensis* and *S. sitiens* (Zumpt, 1973).

The repeatability index, calculated using Procrustes analysis of variance (ANOVA), was used to assess the precision and error associated with the repeatability of outline digitization (Klingenberg and McIntyre, 1998). To assess repeatability, 10 wing images from each *Stomoxys* species were randomly chosen and digitized twice by the same operator. In this study, if the repeatability index for wing shape fell below 90%, all wing images were re-digitized.

#### 2.3.2 Wing cell R_5_ size and shape analyses

Elliptic Fourier analysis (EFA) was used to characterize the size and shape of the contour (Kuhl and Giardina, 1982). Wing cell R_5_ size was estimated based on the perimeter of the contour. One-way ANOVA was used to compare the R_5_ perimeters between the two species, followed by a nonparametric permutation test (1000 permutations) and a Bonferroni correction to determine statistical significance at a *P*-value < 0.05.

Procrustes superposition graphics, using estimated coordinates, were generated to visually compare R_5_ shape among species. The contour shape was quantified using Normalized Elliptic Fourier (NEF) coefficients, which were then subjected to principal component analysis (PCA) to derive principal components (PCs). The PCs from NEF were used as final shape variables for discriminant analysis (DA), and the results were visualized using a factor map. A nonparametric permutation test (1000 permutations) and a Bonferroni correction were used to assess the statistical significance of the Mahalanobis distance at a *P*-value < 0.05.

#### 2.3.3 Unsupervised and supervised classification

In this study, we used unsupervised and supervised classification methods. The unsupervised method aimed to discover natural groupings or patterns in the morphometric data, while the supervised method sought to evaluate the effectiveness of the data in facilitating species identification.

The K-means algorithm, a classical unsupervised method, is widely used for clustering data without the need of individual labels (Chotelersak et al., 2024). In this analysis, we calculated the Euclidean distance using shape variables and set the K parameter to 2 for the K-means algorithm. We then compared the composition of the resulting clusters with the pre-established groups (by species or sex), allowing us to calculate the percentage of correct classification.

For supervised classification, a validated (cross-checked) classification using two methods was used: the Maximum Likelihood method for size (Dujardin et al., 2017) and the DA method based on Mahalanobis distance for shape (Manly, 2004). Both methods were validated using the leave-one-out method (Manly, 2004). The classification models were configured with two classes in separate runs. The 38 PCs were used as input for the DA in each run.

#### 2.3.4 Allometry

The allometric effect, which represents the influence of R_5_ size on R_5_ shape variation, was estimated by calculating the coefficient of determination (*r*^2^) from the regression of the first discriminant factor (DF) of shape against the perimeter.

#### 2.3.5 Software

The contour digitization, data processing, and analysis, as well as the generation of graphical output, were conducted using XYOM (XY Online Morphometrics) version 3, available at https://xyom.io/(Dujardin and Dujardin, 2019).

## 3 Results

### 3.1 Repeatability

The measurements taken by the same user across two sets of images demonstrated high repeatability in shape. The repeatability index score was 95%, with a measurement error of 3%.

### 3.2 Wing cell R_5_ size variation

**Fig. 4:**
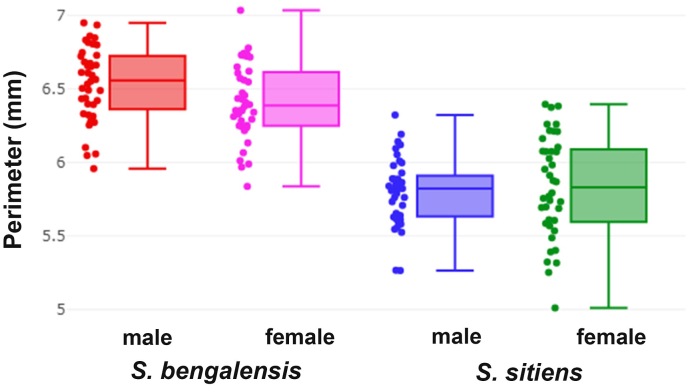
Boxplots for the perimeter of wing cell R_5_ contours for male and female *Stomoxys bengalensis* and *Stomoxys sitiens*. Dots represent the perimeter values for each specimen. The horizontal line crossing each box represents the median, which divides the data between the 25th and 75th quartiles.

The boxplots in Fig. 4 illustrate the perimeters of wing cell R_5_ contours in male and female *S. bengalensis* and *S. sitiens*. The means for male and female *S. bengalensis* were 6.53 mm and 6.41 mm, respectively, while those of male and female *S. sitiens* were 5.80 mm and 5.83 mm, respectively. Both males and females of *S. bengalensis* exhibited significantly larger R_5_ sizes than those of *S. sitiens*. There was no statistically significant difference in R_5_ size between males and females within each species (Table 2).

**Table 2:** Perimeter of the wing cell R_5_ contour in male and female *Stomoxys bengalensis* and *Stomoxys sitiens*, showing statistically significant differences.

Species	Sex	*n*	Mean (mm)	Min-Max	Variance	SD
*S. bengalensis*	Male	40	6.53^A^	5.96–6.95	0.06	0.25
	Female	40	6.41^A^	5.84–7.03	0.07	0.26
*S. sitiens*	Male	40	5.80^B^	5.27–6.32	0.05	0.23
	Female	40	5.83^B^	5.01–6.40	0.12	0.35

*Note*: Statistically significant differences in perimeter (*P* < 0.05) are indicated by different superscript letters.

*Abbreviation*: SD, standard deviation.

### 3.3 Wing cell R_5_ shape variation

**Fig. 5:**
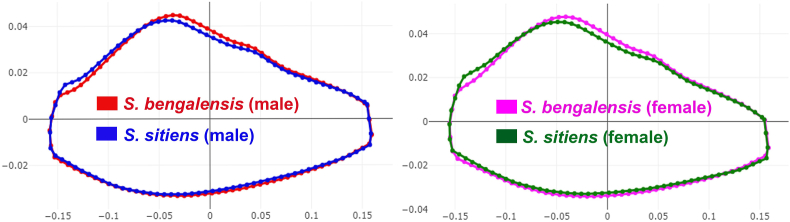
Superposition of wing cell R_5_ contours between *Stomoxys bengalensis* and *Stomoxys sitiens*.

**Fig. 6:**
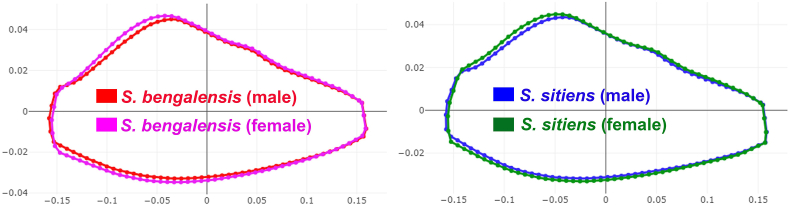
Superposition of wing cell R_5_ contours between male and female *Stomoxys bengalensis* and *Stomoxys sitiens*.

**Fig. 7:**
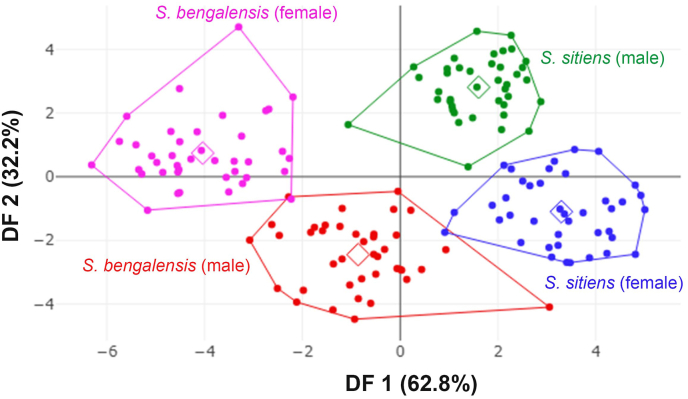
Factor map of the first two discriminant factors shows shape variations between species and sexes of *Stomoxys bengalensis* and *Stomoxys sitiens*. Each point represents an individual, while each polygon corresponds to a different combination of species and sex. Squares indicate the mean values within each group.

The superposition of mean wing cell R_5_ contours revealed areas of difference between species (Fig. 5) and sexes (Fig. 6). The factor map of the first two discriminant factors (DFs) clearly depicted a separation between the species: male *S. bengalensis vs* male *S. sitiens* and female *S. bengalensis vs* female *S. sitiens*) (Fig. 7).

The pairwise Mahalanobis distances of R_5_ shape showed highly significant differences between species and sexes (*P* < 0.001). These distances ranged from 4.52 (between male and female *S. sitiens*) to 7.56 (between female *S. bengalensis* and male *S. sitiens*) (Table 3).

**Table 3:** Mahalanobis distances for wing cell R_5_ shape differences between species and sexes of *Stomoxys bengalensis* and *Stomoxys sitiens* (below diagonal), accompanied by *P*-values (above diagonal).

Species	*S. bengalensis* (male)	*S. bengalensis* (female)	*S. sitiens* (male)	*S. sitiens* (female)
*S. bengalensis* (male)	–	< 0.001	< 0.001	< 0.001
*S. bengalensis* (female)	4.77	–	< 0.001	< 0.001
*S. sitiens* (male)	4.72	7.56	–	< 0.001
*S. sitiens* (female)	5.81	6.15	4.52	–

#### 3.3.1 Unsupervised classification

The classification accuracy based on R_5_ shape, achieved using K-means clustering, yielded high scores for distinguishing between species and sexes of both *S. bengalensis* and *S. sitiens*, with scores ranging from 85% to 95% (Table 4).

**Table 4:** Accuracy scores for distinguishing between species and sexes of *Stomoxys bengalensis* and *Stomoxys sitiens* based on wing cell R_5_ shape, using K-means classification.

Species	Assigned/Observed	Accuracy (%)
Between species		
*S. bengalensis* (male) *vs S. sitiens* (male)	69/80	86.3
*S. bengalensis* (female) *vs S. sitiens* (female)	72/80	90.0
Between sexes		
*S. bengalensis* (male) *vs S. bengalensis* (female)	76/80	95.0
*S. sitiens* (male) *vs S. sitiens* (female)	68/80	85.0

#### 3.3.2 Supervised classification

The accuracy scores for R_5_ size-based classification using a validated method were satisfactory between species but ineffective between the sexes of both *S. bengalensis* and *S. sitiens* (Table 5). The R_5_ shape-based classification, using a validated classification, achieved very high accuracy scores in distinguishing between species and sexes of both *S. bengalensis* and *S. sitiens*, with scores ranging from 90.0% to 97.5% (Table 6).

**Table 5:** Accuracy scores for distinguishing between species and sexes of *Stomoxys bengalensis* and *Stomoxys sitiens* based on wing cell R_5_ size, using a validated classification.

Species	Assigned/Observed	Accuracy (%)
Between species		
*S. bengalensis* (male) *vs S. sitiens* (male)	73/80	91.3
*S. bengalensis* (female) *vs S. sitiens* (female)	66/80	82.5
Between sexes		
*S. bengalensis* (male) *vs S. bengalensis* (female)	51/80	63.8
*S. sitiens* (male) *vs S. sitiens* (female)	40/80	50.0

**Table 6:** Accuracy scores for distinguishing between species and sexes of *Stomoxys bengalensis* and *Stomoxys sitiens* based on wing cell R_5_ shape, using a validated classification.

Species	Assigned/Observed	Accuracy (%)
Between species		
*S. bengalensis* (male) *vs S. sitiens* (male)	73/80	91.3
*S. bengalensis* (female) *vs S. sitiens* (female)	78/80	97.5
Between sexes		
*S. bengalensis* (male) *vs S. bengalensis* (female)	72/80	90.0
*S. sitiens* (male) *vs S. sitiens* (female)	78/80	97.5

### 3.4 Allometry

The analysis of how R_5_ size contributes to R_5_ shape variation between species and between sexes in *S. bengalensis* and *S. sitiens* revealed that size was strongly correlated with shape variation between species. However, the correlation of size with shape variation between sexes was very low (Table 7).

**Table 7:** Linear determination coefficient (allometric effect) between species and sexes of *Stomoxys bengalensis* and *Stomoxys sitiens*.

Species	LDC (%)
Between species	
*S. bengalensis* (male) *vs S. sitiens* (male)	69.3
*S. bengalensis* (female) *vs S. sitiens* (female)	50.7
Between sexes	
*S. bengalensis* (male) *vs S. bengalensis* (female)	6.8
*S. sitiens* (male) *vs S. sitiens* (female)	0.1

*Abbreviation*: LDC, linear determination coefficient.

## 4 Discussion

This study assessed the effectiveness of the wing cell contour, specifically the contour of the first posterior cell (R_5_), in distinguishing between two closely related species within the genus *Stomoxys*, *S. bengalensis* and *S. sitiens*, using outline-based GM. Our results suggest that the wing cell R_5_ contour is promising for distinguishing between these fly species. The effectiveness of using wing contour, including both external and internal wing structures, to differentiate among species has been reported in various insect vectors such as mosquitoes (Chonephetsarath et al., 2021; Laojun et al., 2024), triatomine bugs (Dujardin et al., 2014), and tsetse flies (Kaba et al., 2017). For *Stomoxys* flies, the external contour of the wing has been shown to differentiate among three species, *S. pullus*, *S. uruma*, and *S. indicus*, with accuracy scores ranging from 77% to 96% (Changbunjong et al., 2016a). In the present study, we used only a single internal cell for outline-based GM analysis because it contains the taxonomic characteristics necessary to distinguish between species, as described by Zumpt (1973). Although *S. bengalensis* and *S. sitiens* were easily identified using taxonomic keys, the specimens in this case needed to exhibit distinct morphological characteristics. Identification became challenging when dealing with ambiguous specimens or those lacking key morphological features, especially in the absence of an expert taxonomist for these flies. Recently, landmark-based GM has been reported as highly effective in distinguishing between these flies based on their wing shapes (Changbunjong et al., 2023). However, this method requires several landmarks at the intersections of wing veins and wing boundaries, necessitating a relatively intact wing. This implies that landmark-based GM analysis requires the entire wing for the study. In contrast, outline-based GM can utilize only a single wing cell, making it suitable even for damaged wings, particularly in *Stomoxys* spp. that are prone to damage at the costal margin of the wing during fly collection.

Repeatability and measurement error are crucial considerations in GM analysis (Arnqvist and Martensson, 1998). The measurements taken by the same user across the two sets of images showed a remarkable level of consistency in shape, as evidenced by a high repeatability index score of 95%. Additionally, the margin of error in these measurements was minimal, at only 5%. This underscores the reliability and precision of the measurement process, indicating a high degree of confidence in the results obtained.

The boxplot analysis of perimeter measurements in *S. bengalensis* and *S. sitiens* reveals significant interspecific differences in R_5_ size, with that of *S. bengalensis* being generally larger than *S. sitiens* in males and females. These results corroborate previous research indicating interspecific variation in wing size among different species and populations of *Stomoxys* flies (Changbunjong et al., 2016a; Chaiphongpachara et al., 2022b). The observed differences in R_5_ size between *S. bengalensis* and *S. sitiens* are consistent with well-documented patterns of morphological characteristics, particularly in terms of body length (Masmeatathip et al., 2006; Tumrasvin and Shinonaga, 1978; Zumpt, 1973). Therefore, R_5_ size can be used to distinguish between *S. bengalensis* and *S. sitiens*, with satisfactory accuracy rates, ranging from 82.5% to 91.3%. However, environmental factors such as larval density and substrate quality significantly influence size variation in *Stomoxys* species (Baleba et al., 2019). The lack of significant R_5_ size differences between males and females within each species is consistent with findings from other *Stomoxys* species, including *S. pullus*, *S. uruma*, *S. indicus* (Changbunjong et al., 2016a), and *S. calcitrans* (Changbunjong et al., 2023). This suggests that sexual size dimorphism may not be a significant characteristic in these flies. These results are further supported by the low accuracy scores from R_5_ size-based supervised classification between sexes.

The factor maps produced by the two DFs clearly illustrate a pronounced separation between species, particularly evident in the comparison of male *S. bengalensis* with male *S. sitiens* and female *S. bengalensis* with female *S. sitiens*. The pairwise Mahalanobis distances of R_5_ shape further supported these observations, revealing highly significant differences between species and between sexes. These findings align with previous studies that highlight wing shape differentiation between species and sexes (sexual shape dimorphism) within the genus *Stomoxys* (Changbunjong et al., 2016a, 2023). These results are also supported by the superposition of wing cell contours and the clear external morphological differences observed in wing venation patterns. The observed overlap, although minimal, suggests potential morphological similarities between sexes and species, possibly influenced by environmental and genetic factors (Dujardin, 2008). The allometric effects observed in our study may provide evidence of environmental influences on R_5_ shape. Phylogenetic analyses of mitochondrial and nuclear data for the genus *Stomoxys* revealed that *S. bengalensis* is genetically closer to *S. sitiens* than to other *Stomoxys* species (Dsouli-Aymes et al., 2011). The effectiveness of R_5_ shape-based classification in distinguishing between *S. bengalensis* and *S. sitiens* is demonstrated by high accuracy scores from K-means classification and even higher accuracy scores from R_5_ shape-based supervised classification.

The examination of the allometric effect revealed a strong correlation between R_5_ size and R_5_ shape variation among different species, suggesting that variations in R_5_ size significantly influence R_5_ shape divergence among species. This observation highlights the role of size in determining morphological differences among the distinct species *S. bengalensis* and *S. sitiens*. Conversely, when considering variations in R_5_ shape between sexes within these species, the correlation between size and shape was found to be very low. This suggests that factors such as genetics or sexual dimorphism, beyond just size, play a more prominent role in the shape differences between males and females within the same species. The allometric effect also showed minimal influence between the sexes of other flies within the same subfamily, such as *Haematobosca sanguinolenta* (*r*^2^ = 3%) and *Haematobosca aberrans* (*r*^2^ = 5.7%) (Ardkhongharn et al., 2023).

Overall, the outline-based GM approach, which uses the contour of the internal wing cell, accurately discriminates between *S. bengalensis* and *S. sitiens*. Its performance is nearly equivalent to that of the landmark-based GM approach (Changbunjong et al., 2023), with accuracy scores exceeding 90% and 93%, respectively. Therefore, we recommend this method as an alternative to landmark-based GM when key landmarks for species distinction are missing. Additionally, this method can complement traditional morphological identification when processing ambiguous specimens.

## 5 Conclusions

Our study evaluated the effectiveness of outline-based GM of the first posterior cell of the internal wing to distinguish between species and sexes in two morphologically similar *Stomoxys* species, *S. bengalensis* and *S. sitiens*. The results indicated that the outline-based GM is highly effective in distinguishing between species and sexes of these flies based on the shape of the wing cell contour. Therefore, this method can serve as an alternative to landmark-based GM or complement traditional morphological identification methods for species identification. Correct species identification of these flies is crucial for understanding disease vectors and for developing effective control programmes.

## Funding

This research paper is supported by the Specific League Funds from 10.13039/501100004156Mahidol University.

## Ethical approval

This study protocol was approved by the Animal Care and Use Committee of the Faculty of Veterinary Science at Mahidol University, with the ethics approval number MUVS-2022-01-04.

## CRediT authorship contribution statement

**Thekhawet Weluwanarak:** Conceptualization, Methodology, Investigation, Writing – original draft, Writing – review & editing, Visualization. **Tanawat Chaiphongpachara:** Conceptualization, Methodology, Formal analysis, Investigation, Writing – original draft, Writing – review & editing, Visualization, Supervision. **Tanasak Changbunjong:** Conceptualization, Methodology, Formal analysis, Investigation, Writing – original draft, Writing – review & editing, Visualization, Supervision, Funding acquisition.

## Declaration of competing interests

The authors declare that they have no known competing financial interests or personal relationships that could have appeared to influence the work reported in this paper.

## Data Availability

The data supporting the conclusions of this article are included within the article and its supplementary file.
